# Editorial: New Deputy Editors and Medical Editor

**DOI:** 10.1289/ehp.10984

**Published:** 2007-11

**Authors:** Kenneth S. Korach

**Affiliations:** *EHP*, E-mail: EHPeditor@niehs.nih.gov

In January 2007, *EHP* was fortunate to have Steve Kleeberger and Matt Longnecker step up to serve with me as interim deputy editors in the absence of a full-time permanent editor. Steve and Matt have given generously of their time, expertise, and energy in helping to maintain *EHP* ’s scientific quality during a time of transition. Given *EHP*’s broad scope of coverage, however, we felt the need to likewise broaden the scope of our science editorial expertise. Thus, we are pleased to welcome aboard two additional interim deputy editors.

Michael P. Waalkes is a senior research toxicologist with the National Cancer Institute, where he serves as chief of the Inorganic Carcinogenesis Section, which is part of the Laboratory of Comparative Carcinogenesis stationed at the NIEHS campus in Research Triangle Park, North Carolina. Mike received his PhD in pharmacology and toxicology from West Virginia University. His postdoctoral studies at the University of Kansas School of Medicine focused on the cellular and molecular mechanisms of acquired tolerance to metal toxicity. His current research involves defining the mechanisms of action of the carcinogenic inorganics, including arsenic, lead, and cadmium. Mike is currently an adjunct professor of molecular toxicology at Duke University and an active member of the Society of Toxicology. He has been the editor of *Toxicology and Applied Pharmacology* since 2000 and serves on the editorial boards of *Toxicology*, the *Journal of Toxicology and Environmental Health*, and *Toxicology Mechanisms and Methods*.

Stephanie London is a senior investigator in the NIEHS Epidemiology Branch with a joint appointment in the Laboratory of Respiratory Biology. She received her MD and her DrPH degree in epidemiology from Harvard University. She completed a residency in internal medicine at the Massachusetts General Hospital and a residency in occupational and environmental medicine at Harvard, and is board certified in both fields. Stephanie was an assistant professor at the University of Southern California School of Medicine from 1989 through 1995, where she was part of a small team of investigators who founded a landmark study of health effects of air pollution in schoolchildren known as the Children’s Health Study. She came to the NIEHS in 1995. Her work focuses on genetics and interactions between genetics, diet, and environmental pollutants in relation to asthma and chronic obstructive pulmonary disease. Stephanie is an editor for *Epidemiology* and has served on the editorial board of the *American Journal of Epidemiology*.

I would also like to take this opportunity to introduce our new medical editor, Russ Hauser, who succeeds Brian S. Schwartz. Russ is an associate professor of environmental and occupational epidemiology in the departments of Environmental Health and Epidemiology at the Harvard School of Public Health. He graduated from Clark University and the Albert Einstein College of Medicine, where he received his MD. He received his MPH and ScD from the Harvard School of Public Health, where he completed a residency in occupational medicine. He is board certified in occupational medicine. From 2000 to 2004, he served as deputy director of the Occupational and Environmental Medicine Residency, National Institute for Occupational Safety and Health Education and Research Center. Russ’s research focuses on the effects of environmental and occupational chemicals on fertility and pregnancy outcomes. He is on the editorial board of the *Journal of Exposure Science and Environmental Epidemiology*, and is chair of the Environment and Reproduction Special Interest Group of the American Society for Reproductive Medicine.

Finally, we bid a fond farewell to Brian, our outgoing medical editor. Brian served in this role from July 2004 through July 2007. During this time he significantly raised the bar for *EHP* ’s Grand Rounds and Environmental Medicine submissions, allowing the journal to advance in its mission of delineating relationships between the environment and human health. We are most grateful for his service to the journal.

Stay tuned for news of a permanent editor-in-chief for *EHP*. As always, we welcome your feedback.

## Figures and Tables

**Figure:**
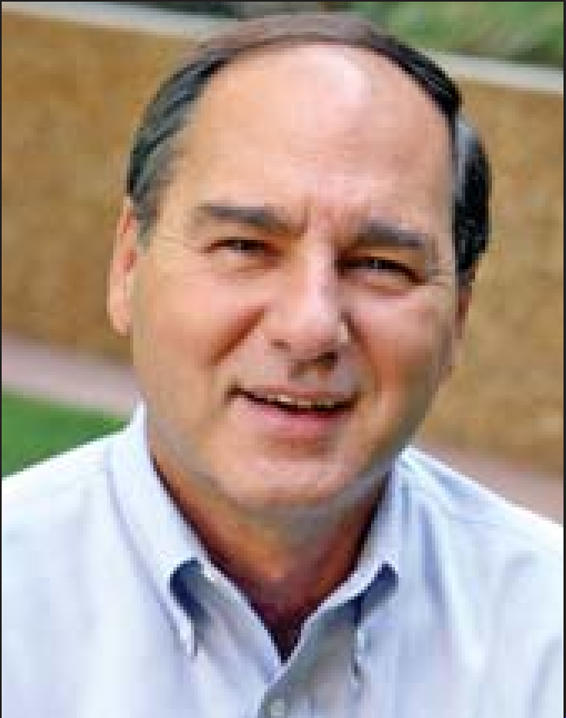
Kenneth S. Korach

**Figure:**
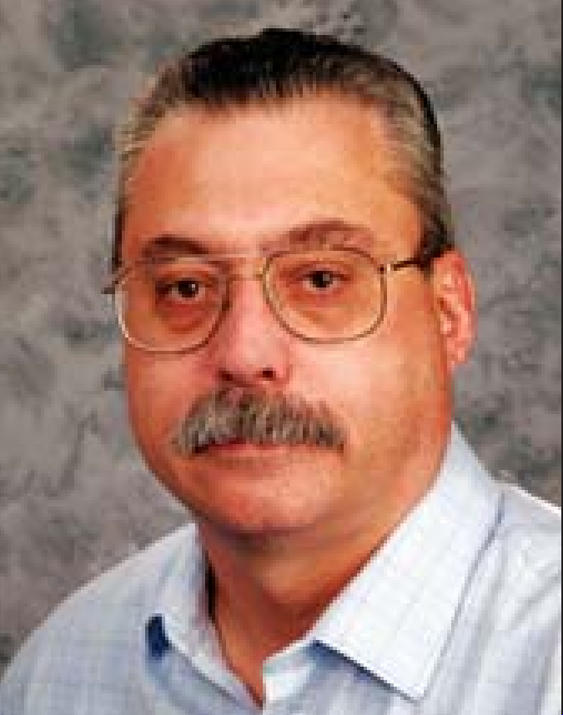
Michael R. Waalkes

**Figure:**
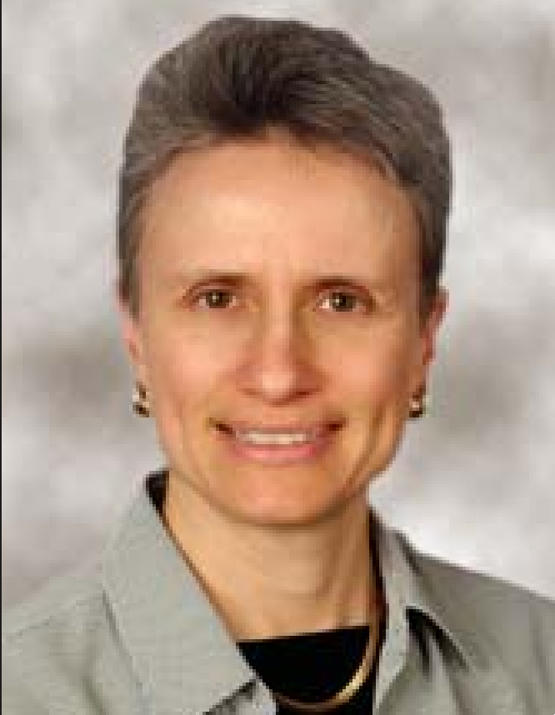
Stephanie London

**Figure:**
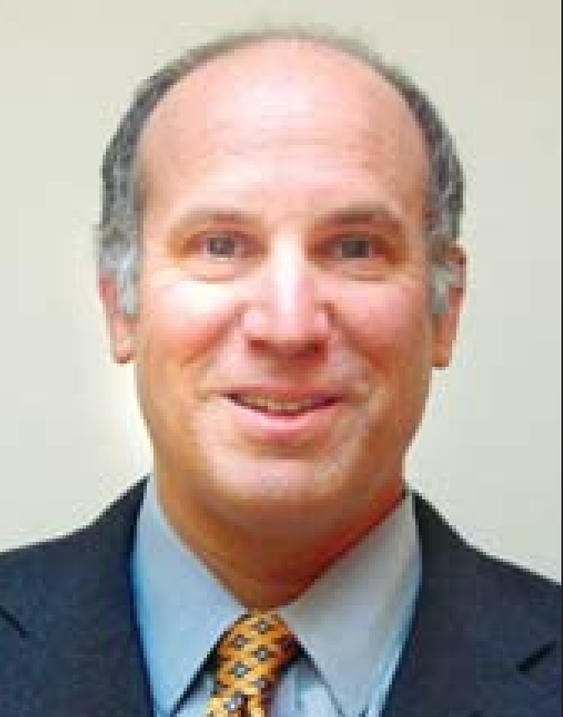
Russ Hauser

